# Supplementary material for: Analysis of hippocampal synaptic function in a rodent model of early life stress
Source: Wellcome Open Res. 2024 Jun 10;9:300. [Version 1] doi: 10.12688/wellcomeopenres.22276.1 (PMC11362746; doi:10.12688/wellcomeopenres.22276.1)
Supplement: Peer review history

# Reviewer report: Reviewer response for version 1

**DOI:** 10.21956/wellcomeopenres.24553.r88872

**Published:** 2024-08-29

**Authors:** Lubica Lacinova

**Affiliations:** 1 Slovak Academy of Sciences, Bratislava, Slovakia

This study is focused on effects of early life stress on hippocampal synaptic function in the early life up to approximately 4 months. Early life stress was evoked by 180 min long maternal separation (MS180) during postnatal days 1-14 (PND 1-14). Research question is clearly formulated and Methods are described in sufficient details. Description of results and experimental design includes several points which should be better explained.

**Major points:**

 1. It is not explained why animals were divided into 2 cohorts. Both cohorts started with the same MS180 model. Second cohort consisted from just 2 female rats and their offspring. For the sake of solid statistics one experimental cohort should have at least 3 litters. Generally, number of rats/offspring does not fit with data in the first paragraph of Methods. 19 females and 82 offspring are listed there. In the Figure 1 only 6 female and 52 offspring are included. Even if 4 females and litters failed, as mentioned in the second paragraph, this will not fit. The authors justify that some experiments were limited by Covid, nevertheless, there was enough time after Covid rules were relaxed to run additional experiments to reach statistical power.

 2. Timing of individual tests is not justified. Validation of the model was done in a late stage while electrophysiology in the first cohort preceded it – why? Also, LTP measured well after AMPA/NMDA experiments, so that results of these experiments cannot be used to explain changes in LTP (or lack thereof). Authors admit that at the age PND 112-123 neurogenesis may be so low that no further changes due to MS180 can be detected – and indeed, no changes were detected. Knowing this, why these test were not done at earlier age?

 3. It is not clear how the experiments demonstrated in the Figure 2 F-I were done. Four groups: male, female, control and MS180 are shown. It seems that only effect of the restraint stress on cFos and BrdU expression was estimated and basal expression in animals unexposed to either MS180 or restraint stress was not investigated. As there was a significant difference between male and female groups in reaction to restrain stress and/or MS180, it is of interest whether a basal expression of both markers does differ between sexes. As shown in the Figure 3, MS180 lowers AMPA/NMDA ratio, but the same effect is observed when AMPA/NMDA ratio is compered between male and female groups.

 4. Discussion mostly interprets data obtained by the authors in comparison with results obtained under similar conditions by other groups. Authors should go a step further and explain, how the changes they observed in MS180 animals may (or may not) explain psychiatric disorders occurring as a consequence of an early life stress.

**Minor points:**

 1. Please include explanation of abbreviations in the legend to the Figure 1.

Is the work clearly and accurately presented and does it cite the current literature?

Partly

If applicable, is the statistical analysis and its interpretation appropriate?

I cannot comment. A qualified statistician is required.

Are all the source data underlying the results available to ensure full reproducibility?

Yes

Is the study design appropriate and is the work technically sound?

Partly

Are the conclusions drawn adequately supported by the results?

Partly

Are sufficient details of methods and analysis provided to allow replication by others?

Yes

Reviewer Expertise:

Electrophysiology

I confirm that I have read this submission and believe that I have an appropriate level of expertise to confirm that it is of an acceptable scientific standard, however I have significant reservations, as outlined above.

# Reviewer report: Reviewer response for version 1

**DOI:** 10.21956/wellcomeopenres.24553.r88868

**Published:** 2024-08-01

**Authors:** Tak Pan Wong

**Affiliations:** 1 McGill University, Montreal, Québec, Canada

- Is the work clearly and accurately presented and does it engage with the current literature?
   
  - Wilkinson et al in this paper examined the effect of maternal separation, a form of early life stress, on the behaviors and hippocampal synaptic function of adult offspring. Early life stress has been associated with increased risk of mood disorders such as depression. As the hippocampus has been highly implicated in stress-related disorders, many studies have investigated the impact of early life stress on hippocampal function, including NMDA receptor that plays crucial roles in synaptic plasticity. Limitations of previous studies include the use of primarily male animals. In addition, most previous studies overlooked the impact of early life stress of different synaptic pathways in the hippocampus. In the current study, the authors used Long-Evans rats to examine the impact of daily 180 minutes maternal separation (MS180) from postnatal day 1 to 14 on adult rats behaviors (anxiety, anhedonia), stress induced corticosterone release, neurogenesis, and hippocampal synaptic functions in brain slices. Briefly, they repeated some of their previous findings that maternal separation increased the feed latency and thigmotaxis in both male and female rats. MS180 also increased stress induced corticosterone release in rats of both sexes. cFos expression, which representing activated neurons, in the PVN of male rats was increased by MS180. Finally, MS180 increased NMDAR currents in both male and female mice, without altering AMPAR currents and LTP in the CA1.
  - Overall, the current study is clearly presented. The authors have discussed most studies that have examined the impact of early life stress on NMDA receptor function. However, an omission was Bagot et al. 2012, who showed that low maternal care as a form of early life stress also increased NMDAR function in the dentate gyrus.

 

- Is the study design appropriate and does the work have academic merit?
   
  - Given that most studies of the impact of early life stress on hippocampal function have focused on male offspring, including female offspring in the current study is a strength. In addition, the effects of early life stress on different pathways were examined to reveal potential differences in the impact of early life stress on these pathways. The design of experiment is appropriate with careful consideration of using similar litter size, reversed light cycle for behavioral experiments. Although no differences were found, examining electrophysiological properties of both dorsal and ventral slices is a strength.
- Are sufficient details of methods and analysis provided to allow replication by others?
   
  - Methods are clearly described and easy to understand. Figures are clear and individual data points are shown in histograms. Schematics for experimental design and the placement of electrodes for ephys recording were provided. All these factors make the replication of the current study feasible.
- If applicable, is the statistical analysis and its interpretation appropriate?
   
  - Statistical analyses are thorough and clearly described. Apart from using regular tests for pairwise and group comparison, multilevel analysis was used to consider the impact of litter and animal on ephys data. Pitfalls and limitations, such as using approaches to avoid pseudoreplication, and the low sample size for LTP experiments due to COVID, were reported.
- Are all the source data underlying the results available to ensure full reproducibility?
   
  - Apart from showing individual data points in all histograms, data such as raw ephys data are available through an open science framework.
- Are the conclusions drawn adequately supported by the results?
   
  - Evidence obtained by the group support the conclusion that NMDAR function was enhanced by early life stress. Although the lack of change of miniature synaptic currents supported no change in AMPAR function, additional AMPARs are likely recruited in evoked EPSCs. Having data of input/output relationship of evoked synaptic responses (e.g. fiber volley vs fEPSP slope) would be stronger evidence to confirm the lack of effect of early life stress on AMPAR function.

Is the work clearly and accurately presented and does it cite the current literature?

Yes

If applicable, is the statistical analysis and its interpretation appropriate?

Yes

Are all the source data underlying the results available to ensure full reproducibility?

Yes

Is the study design appropriate and is the work technically sound?

Yes

Are the conclusions drawn adequately supported by the results?

Yes

Are sufficient details of methods and analysis provided to allow replication by others?

Yes

Reviewer Expertise:

Impact of stress on synaptic and cellular activity in the hippocampus

I confirm that I have read this submission and believe that I have an appropriate level of expertise to confirm that it is of an acceptable scientific standard.

# Reviewer report: Reviewer response for version 1

**DOI:** 10.21956/wellcomeopenres.24553.r87558

**Published:** 2024-08-01

**Authors:** Rafael Naime Ruggiero

**Affiliations:** 1 University of São Paulo, São Paulo, Brazil

The study by Matthew P. Wilkinson and collaborators examined the impact of maternal separation (MS180) on NMDAR function in Hooded Long-Evans rat pups. MS180 animals exhibited increased feeding latency, elevated corticosterone levels, and heightened PVN cFos expression, indicating stress response alterations. Although MS180 rats had a lower AMPA/NMDA ratio, no changes were observed in miniEPSC amplitude, area, or long-term potentiation (LTP), suggesting that increased NMDAR function did not coincide with enhanced LTP. The authors conclude that the MS180 model displayed a behavioral phenotype consistent with previous studies, demonstrating increased NMDAR function without a corresponding increase in LTP.

 The study is straightforward and distinguishes itself from previous maternal deprivation studies by considering key factors such as animal sex and specific hippocampal regions. The study is quite transparent in its results and findings, even noting some tests that were underpowered. Despite this transparency, the presence of underpowered tests casts doubts on the results, creating uncertainty about which findings are significant and which are due to insufficient power. Furthermore, some choices in statistical methodology need better justification, as detailed below.

**Major comments**

 Overall, the study gives the impression of not being adequately planned in statistical terms.

- The authors state that they based the sample size on previous studies. Ideally, the justification for the sample size should mention the desired statistical power (typically 80% or 90%) and the significance level (typically 0.05), as well as the expected effect size. These details are not provided.
- The fact that many tests are underpowered (according to the authors themselves) makes it difficult to interpret the results, especially considering that many findings do not align with previous literature using similar models.
- The authors combined two cohorts of animals (possibly subjected to different light cycle conditions, see comment below) to evaluate a single behavioral test.

Why were different light cycles used for the groups? This needs to be justified. This is mentioned in the methodology, but no explanation is provided for this fact or for the change from one group to another. Additionally, the authors state that "animals from cohort 2 were used to provide additional power in the NSFT experiment," combining animals from cohort 1 and cohort 2. Animals from the validation cohort were subjected to the inverted light cycle; however, the authors do not state whether the same light cycle inversion was applied to cohort 2. This could mean that animals subjected to the same test were exposed to different light-dark cycles.

 Stepwise methods for variable selection may lead to overfitting, biased model selection favoring spurious correlations, unreliable inference, and reduced transparency, highlighting the importance of careful consideration of their limitations in statistical inference. This is particularly problematic in the context of a small sample size and underpowered tests.

**Minor comments**

 In the sentence "However, a major drawback of previous work is that it has not considered the key factors of animal sex, hippocampal region, and input pathway," what do the authors mean by "input pathway" in this context? The term seems vague.

 How was the division of litters into experimental groups done? This is important to avoid specific litter effects on the results of a group.

 Please include in the figure legends the symbols used, such as * or ##.

 Did the authors control for the estrous cycle of the females or measure the phase of the estrous cycle during testing? A significant portion of behavioral variability could result from differences in the estrous cycle, potentially masking the results.

 The authors state that "Overall, while not completely matching previous data, these validation experiments give enough confidence that a phenotype matching that previously seen in other ELS models was created to allow interpretation of electrophysiological data." However, as noted in their own discussion, many differences were found (in fact, there appear to be more differences than similarities with previous studies). What criteria were used to assert that a "phenotype matching that previously seen in other ELS models was created"?

 Another, more epistemological, point is that behavioral patterns using the same or similar models are observed in different studies, which may be related to changes in protocol, different animal strains, underpowered positive results that are not replicable, or even variability in response due to the model having more subtle effects. Given this great variability, I question whether it is more appropriate to investigate the neurobiology of specific behaviors induced by the models rather than attempting to replicate a general phenotype associated with general neurobiological alterations, which is often highly variable.

Is the work clearly and accurately presented and does it cite the current literature?

Yes

If applicable, is the statistical analysis and its interpretation appropriate?

Partly

Are all the source data underlying the results available to ensure full reproducibility?

No

Is the study design appropriate and is the work technically sound?

Yes

Are the conclusions drawn adequately supported by the results?

Yes

Are sufficient details of methods and analysis provided to allow replication by others?

Yes

Reviewer Expertise:

Neuroscience, electrophysiology, animal models of psychiatric symptoms, early-life seizures.

I confirm that I have read this submission and believe that I have an appropriate level of expertise to confirm that it is of an acceptable scientific standard, however I have significant reservations, as outlined above.
